# *CYP19* Genetic Polymorphism Haplotype *AASA* Is Associated with a Poor Prognosis in Premenopausal Women with Lymph Node-Negative, Hormone Receptor-Positive Breast Cancer

**DOI:** 10.1155/2013/562197

**Published:** 2013-11-14

**Authors:** Sung-Hsin Kuo, Shi-Yi Yang, Huang-Chun Lien, Chiao Lo, Ching-Hung Lin, Yen-Sen Lu, Ann-Lii Cheng, King-Jeng Chang, Chiun-Sheng Huang

**Affiliations:** ^1^Department of Oncology, National Taiwan University Hospital and National Taiwan University College of Medicine, Taipei, Taiwan; ^2^Department of Internal Medicine, National Taiwan University Hospital and National Taiwan University College of Medicine, Taipei, Taiwan; ^3^Graduate Institute of Oncology, National Taiwan University College of Medicine, Taipei, Taiwan; ^4^Genomics Research Center, Academia Sinica, Taipei, Taiwan; ^5^Graduate Institute of Epidemiology, College of Public Health, National Taiwan University, Taipei, Taiwan; ^6^Department of Pathology, National Taiwan University Hospital and National Taiwan University College of Medicine, Taipei, Taiwan; ^7^Department of Surgery, National Taiwan University Hospital, No. 7, Chung-Shan South Rd, Taipei 100, Taiwan

## Abstract

Given the critical role of *CYP19* in estrogen synthesis, we investigated the influence of *CYP19* gene polymorphisms on the clinical outcome of lymph node- (LN-) negative, hormone receptor- (HR-) positive early breast cancers. Genotyping for the *CYP19* polymorphisms rs4646 (A/C), rs1065779 (A/C), *CYP19* (TTTA)n (short allele/long (S/L) allele using the 7 TTTA repeat polymorphism as the cut-off), and rs1870050 (A/C) was performed on 296 patients with LN-negative, HR-positive breast cancers. All patients received adjuvant hormonal therapy. Associations were examined between these 4 genotypes and 6 common haplotypes of *CYP19* and distant disease-free survival (DDFS), disease-free survival (DFS), and overall survival (OS). Patients were divided into the 6 subhaplotypes of *CCLA* (41.1%), *AASA* (17.1%), *CASA* (11.9%), *CCLC* (8.9%), *CCSA* (7.5%), *AASC* (8.9%), and others (4.6%). In premenopausal patients, haplotype *AASA* was significantly associated with a poor DDFS (adjusted hazard ratio (aHR), 3.3; *P* = 0.001), DFS (aHR, 2.5; *P* = 0.0008), and OS (aHR, 2.9; *P* = 0.0004) after adjusting for age, tumor size, tumor grade, estrogen receptor status, progesterone receptor status, chemotherapy, pathology, adjuvant hormone therapy, menopausal status, and radiotherapy. Furthermore, haplotype *AASA* remained a negative prognostic factor for premenopausal patients receiving adjuvant chemotherapy in terms of DDFS (aHR, 4.5; *P* = 0.0005), DFS (HR, 3.2; *P* = 0.003), and OS (HR, 6.4; *P* = 0.0009). However, in postmenopausal patients, haplotype *AASA* was not associated with a poor prognosis, whereas the *AASC* haplotype was significantly associated with a poor DFS (aHR, 3.1; *P* = 0.03) and OS (aHR, 4.4; *P* = 0.01). Our results indicate that, in patients with LN-negative, HR-positive breast cancers, genetic polymorphism haplotype *AASA* is associated with poor survival of premenopausal women but does not affect survival of postmenopausal women.

## 1. Introduction

For estrogen receptor- (ER-) or progesterone receptor- (PR-) positive breast cancers, hormone-based treatment, such as ovarian ablation, tamoxifen, or aromatase inhibitors, has been demonstrated to result in an improvement in disease-free survival (DFS) and overall survival (OS) [1, 2]. Given the critical role of aromatase in estrogen synthesis, it is important to study the relationship between activity of aromatase, encoded by gene *CYP19*, a single copy gene located on chromosome 15q21.2 [3–5], and prognosis of breast cancer patients receiving adjuvant hormone treatment. In a nested case-control study evaluating the associations between *CYP19 *repeat polymorphisms and breast cancer risk, Haiman et al. [6] showed that women with the 8-repeat allele of the TTTA polymorphism have higher estrogen levels than those with the 7-repeat allele. One population-based study also showed that a higher repeat number of the TTTA repeat polymorphism is associated with a longer survival of breast cancer patients [7].

Our previous study demonstrated that, in stages I-II and operable stage III breast cancer patients, hormone receptor- (HR-) positive premenopausal patients with the long allele of the *CYP19* polymorphism have a significantly better DFS and OS than those without the long allele, but this prognostic effect of the *CYP19 *polymorphism is not seen in postmenopausal women patients [8]. A recent whole genome analysis of the human *CYP19* gene using DNA samples from four ethnic groups, including Han Chinese-Americans, identified at least 88 single nucleotide polymorphisms (SNPs) and 44 common haplotypes and demonstrated that there were 30 large ethnic variations in both allele frequencies and haplotypes and that 6 polymorphisms were observed only in Han Chinese-American subjects [9]. From this database and a literature review, we selected the* CYP19 *TTTA repeat and three *CYP19* SNPs, rs4646 in the 3′ untranslated region, rs1065779 in the introexon boundary, and rs1870050 in the promoter region/untranslated exon [9–14], which occur at a higher frequency in Han Chinese (see Supplementary Table 1 in the Supplementary Material available online at http://dx.doi.org/10.1155/2013/562197), are associated with cancer risk [9–12], and are suggested to influence aromatase function [13, 14].

Since haplotype analysis can capture the cisacting causal variants that are potentially associated with disease risk and disease progression [15–17], we investigated the effect of 6 haplotypes of these SNPs in *CYP19* on the clinical outcome of LN-negative, HR-positive early breast cancers. 

## 2. Methods

### 2.1. Study Cohort and Sources of Information

Eligible women were newly diagnosed patients with stage I or II (AJCC 2007) LN-negative, HR-positive early breast cancers diagnosed at the National Taiwan University Hospital between January 1, 1994, and June 30, 2004. All data on the histological grade and HR status of the primary tumors were reviewed by one pathologist (Dr. Lien). Patients were considered HR-positive if the percentage of ER- or PR-positive epithelial cells was ≥ 10%; 44.5% (132/296) of the included patients had taken part in our previous molecular epidemiological study [8]. Genomic DNA and detailed demographic information were obtained from the patients and their medical charts with their consent. The pathologic review, archiving of tumor tissues and blood samples, and genetic studies were approved by the institutional review board of the National Taiwan University Hospital. Pathologic and clinical information about treatment (including type of surgery, receipt or nonreceipt of adjuvant systemic therapy, and type and dose of adjuvant systemic therapy) and follow-up information (including recurrence and distant metastasis) were obtained from the pathology reports and clinical records. If menstruation had taken place within one year, the woman was considered as premenopausal, and, if not, as postmenopausal. Women who had undergone hysterectomy, but without bilateral oophorectomy, were considered as premenopausal if they were younger than 52 and as postmenopausal if older. 

Patients with high-risk factors, for example, grade III cancers and large tumors, but receiving nonstandard adjuvant chemotherapy, as defined in our previous study [18], were excluded, since our previous study demonstrated that breast cancer patients receiving standard adjuvant chemotherapy have a better DFS and OS than those receiving nonstandard adjuvant chemotherapy [18]. In the present study, the definition of standard adjuvant treatment was based on whether the indication and the regimen and dose of adjuvant chemotherapy were the same as those in the literature or those recommended by the NCCN guidelines, NIH consensus, and St. Gallen consensus [18–20]. All enrolled patients received adjuvant hormonal therapy. Adjuvant radiotherapy was administered to all patients after breast conserving surgery [20, 21]. All patients receiving postoperative radiotherapy received an optimal dose of radiation (a biologically equivalent dose of 50–60 Gy in 2-Gy fractions) [20, 21]. After surgery and adjuvant therapy, the patients were regularly followed up in our clinic. If patients were lost to followup, information on disease status and survival was obtained from the patients' charts, hospital cancer registry records, and the National Death Registry.

### 2.2. DNA Isolation and Aromatase Genotyping

*CYP19 *genotyping for rs4646 (A/C), rs1065779 (A/C), (TTTA)n, and rs1870050 (A/C) was performed on 296 Taiwanese patients with LN-negative, HR-positive breast cancers. A sample of peripheral blood, collected in acetate-citrate dextrose, was obtained from each patient and the buffy coat immediately prepared and stored at −80°C until extraction of genomic DNA. Genomic DNA was extracted using a conventional proteinase K extraction and a QIAamp DNA Blood Kit (Qiagen, Valencia, CA) following the manufacturer's protocol and stored at −80°C.

The TTTA tetranucleotide repeats of *CYP19* were identified using the polymerase chain reaction and electrophoresis in an ABI PRISM 3130XL, as described previously [8]. TaqMan assays were used for genotyping of rs4646 (A/C), rs1065779 (A/C), and rs1870050 (A/C). The thermal cycling conditions were 50°C for 2 minutes and 95°C for 10 minutes, followed by 40 cycles of 95°C for 15 seconds and 60°C for 60 seconds. The PCR reaction was performed in a total reaction volume of 5 *μ*L containing 10 ng of genomic DNA, 2.5 *μ*L of 2x TaqMan Universal PCR Master Mix (Applied Biosystems), and 0.125 *μ*L of the 40X primers/probes in a 384-well plate format on an ABI7900HT. Genotyping was performed as a Made to Order Assay (Applied Biosystems: C_8234730_1_ for rs4646, C_8234755_10 for rs1065779, and C_11672268_20 for rs1870050).

### 2.3. Statistical Analysis

The effect of genotype was initially evaluated using a codominant model in which each genotype was considered separately. However, the estimated HRs (hazard ratios) for each genotype were imprecise because of the small number of cases, so some of the genetic polymorphisms were classified into 2 groups by pooling the heterozygous group with either the homozygous variant or wild-type group on the basis of the estimated odds ratio of the heterozygous genotype (poor survival or good survival). The TTTA repeat in intron 4 of *CYP19* was dichotomized as S/L (one short allele ≤ 7 repeats and one long allele > 7 repeats) and L/L (two long alleles > 7 repeats) versus S/S (two short alleles ≤7 repeats). Furthermore, based on the aforementioned observed genotype data, several individual genotypes were combined for analysis into *haplotypes* using PROC *HAPLOTYPE *in *SAS *Genetics, as in Tan's study [22]. Haplotypes with a probability less than 0.9 were not included in the subsequent association analysis. Of the 11 haplotypes constructed, those with an estimated frequency of an individual allele less than 5% were not included in the association analyses because of limited sample size and heterogeneity.

Follow-up data available as of December 31, 2010, were analyzed. Distant disease-free survival (DDFS) was measured from the date of the original surgery for breast cancer to distant recurrence or death from any cause, disease-free survival (DFS) was measured from the date of the original surgery for breast cancer to the date of locoregional or distant recurrence or death from any cause, and overall survival (OS) was measured from the date of the original surgery to the date of death from any cause or the last follow-up date [23]. Survival was calculated using the product limit method of Kaplan and Meier. Differences in survival were compared between groups using the log-rank test. 

The hazard ratio and the corresponding 95% confidence interval (CI) for each variable were estimated using Cox regression analyses. The multivariate-adjusted hazard ratio (aHR) of progression associated with the individual genotypes and haplotypes of the screened SNPs was assessed for the total patient group after adjusting for age, tumor size, tumor grade, ER and PR status, chemotherapy, pathology, adjuvant hormone therapy, menopausal status, and radiotherapy and for the pre- and postmenopausal groups by adjusting for age, tumor size, tumor grade, ER and PR status, chemotherapy, pathology, adjuvant hormone therapy, menopausal status, and radiotherapy stratified by menopausal status. Two-sided *P* values less than 0.05 were considered statistically significant. All analyses were performed using SAS statistical software for Windows version 9.2 (SAS Institute, Cary, NC, USA).

## 3. Results

### 3.1. Clinicopathologic Features

Two hundred and ninety-six patients were included in the study. As shown in Table 1, the median age was 50 years (range 24–81 years), and 178 subjects were premenopausal and 118 postmenopausal. The clinicopathologic characteristics and treatments are also listed in Table 1. Briefly, all ER-positive and/or PR-positive patients received tamoxifen, and some (9.1%) received ovarian ablation or a luteinizing hormone-releasing hormone agonist with or without tamoxifen. None of the patients received aromatase inhibitors as adjuvant hormonal therapy. One hundred and thirty-four (45.3%) received adjuvant standard chemotherapy and 162 (54.7%) did not. 

### 3.2. *CYP19 *Polymorphisms and Prognosis

As shown in Table 2, based on the analysis of all patients, the *rs4646* polymorphism (A/A and A/C genotypes versus the C/C genotype) was marginally associated with a poor DFS (aHR 1.6.; 95% CI, 1.0–2.6; *P* = 0.06) and a poor DDFS (aHR, 1.7; 95% CI, 0.9–2.9; *P* = 0.07). After stratification by menopausal status, in premenopausal patients, it was marginally associated with a poor OS (aHR, 2.4; 95% CI, 1.0–5.9; *P* = 0.06) and poor DFS (aHR, 1.8; 95% CI, 0.9–3.2; *P* = 0.07) and was significantly associated with a poor DDFS (aHR, 2.3; 95% CI, 1.1–4.9; *P* = 0.02). However, in postmenopausal women, this SNP was not associated with OS, DFS, or DDFS. 

In the whole group, the *rs1065779* polymorphism (A/A and A/C genotypes versus the C/C genotype) was significantly associated with a poor DFS (aHR, 2.3; 95% CI, 1.2-4.2; *P* = 0.009) and marginally associated with a poor DDFS (aHR, 1.9; 95% CI, 1.0–3.7; *P* = 0.06) but was not associated with OS. After stratification by menopausal status, in premenopausal patients, it was significantly associated with a poor DFS (aHR, 3.5; 95% CI, 1.5–7.9; *P* = 0.003) and poor DDFS (aHR, 3.2; 95% CI, 1.2–8.6; *P* = 0.02) but not a poor OS. In contrast, in postmenopausal women, this SNP was not associated with a poor prognosis (OS, DFS, or DDFS).

In the whole group, the TTTA repeat polymorphism in intron 4 of *CYP19* (S/S and S/L genotypes versus the L/L genotype) was significantly associated with a poor progression in terms of OS (aHR, 3.3; 95% CI, 1.0–11.0; *P* = 0.05), DFS (aHR, 2.8; 95% CI, 1.3–6.2; *P* = 0.01), and DDFS (aHR, 2.8; 95% CI, 1.1–7.1; *P* = 0.03). After stratification, this SNP remained a negative prognostic factor in premenopausal patients in terms of OS (aHR, 7.8; 95% CI, 1.0–58.9; *P* = 0.05), DFS (aHR, 4.0; 95% CI, 1.4–11.4; *P* = 0.009), and DDFS (aHR, 5.5; 95% CI, 1.3–23.5; *P* = 0.02) but was not associated with a poor prognosis of OS, DFS, and DDFS in postmenopausal women.

The rs1870050 polymorphism (A/A genotype versus A/C and C/C genotypes) was significantly associated with a poor DDFS in both the whole group (aHR, 1.8; 95% CI, 1.0–3.1; *P* = 0.05) and premenopausal women (aHR, 2.1; 95% CI, 1.0–4.3; *P* = 0.05). However, no significant associations were observed for OS or DFS. The polymorphism was not associated with poor prognosis (OS, DFS, or DDFS) in postmenopausal women. 

In premenopausal patients with adjuvant chemotherapy (high-risk group), the *rs1065779* and rs1870050 polymorphisms were significantly associated with a poor prognosis in terms of DDFS (aHR, 13.0; 95% CI, 1.6–104.8; *P* = 0.02 and aHR, 3.3; 95% CI, 1.0–10.7; *P* = 0.05, resp.), but no associations were seen for the *rs4646 *and TTTA repeat polymorphisms. 

### 3.3. *CYP19 *Haplotypes and Prognosis

Since SNPs tend to be inherited in clusters and linked to the same functional variants, which are potentially associated with disease prognosis [15–17], the 4 genetic polymorphisms of *CYP19* were clustered into haplotypes of *CCLA* (41.1%), *AASA* (17.1%), *CASA* (11.9%), *CCLC* (8.9%), *CCSA* (7.5%), *AASC* (8.9%), and others (4.6%). There were no significant differences in clinicopathologic features, adjuvant hormone treatment (tamoxifen versus ovarian ablation and LHRH agonist), or systemic adjuvant chemotherapy (no versus yes) between these groups (data not shown).

**Figure 1:**
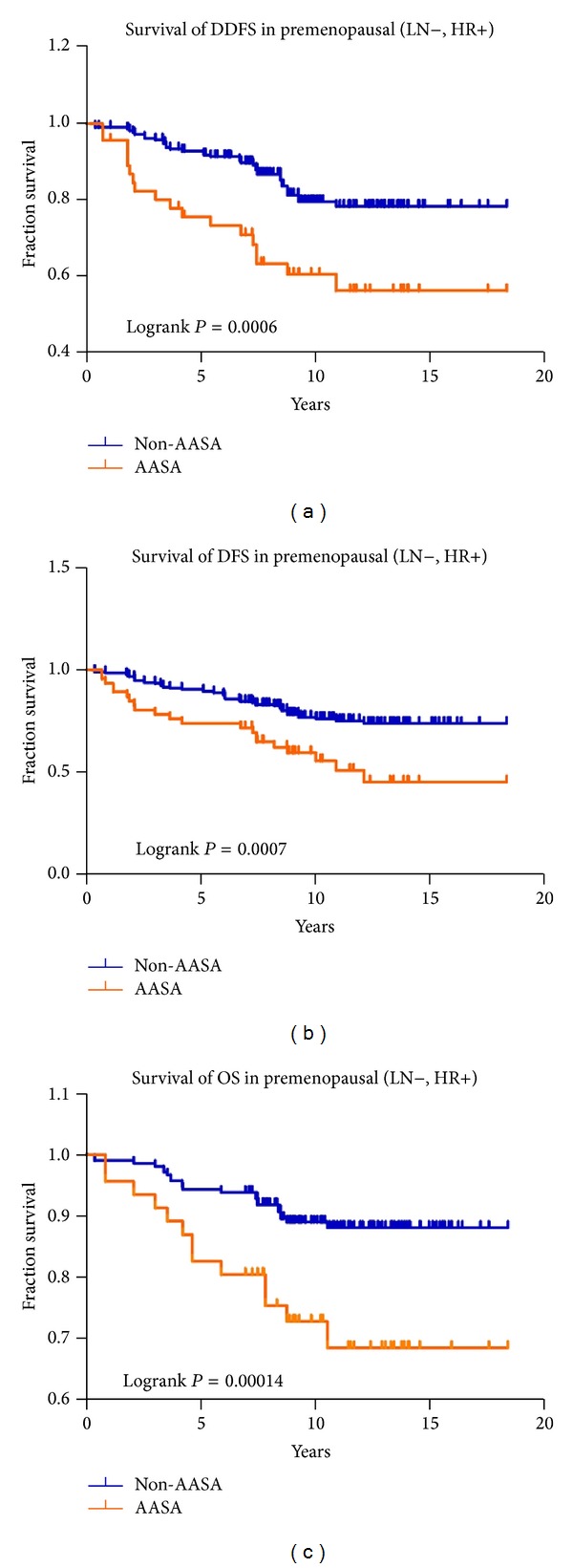
Overall treatment results for LN-negative, HR-positive premenopausal patients as a function of *CYP19* haplotype (*AASA* haplotype versus non-*AASA *haplotype) for (a) distant disease-free survival (DDFS), (b) disease-free survival (DFS), and (c) overall survival (OS).

As shown in Table 3, taking all patients together, the haplotype subtype *AASA* was found to be associated with a poor OS (aHR, 2.1; 95% CI, 1.2–3.9; *P* = 0.01), DFS (aHR, 2.0; 95% CI, 1.3–3.2; *P* = 0.002), and DDFS (aHR, 2.0; 95% CI, 1.3–3.2; *P* = 0.008) compared to non-*AASA* alleles. After stratification by menopausal status, in premenopausal patients, haplotype *AASA* remained associated with a poor prognosis in terms of OS (aHR, 3.3; 95% CI, 1.6–6.8; *P* = 0.001), DFS (aHR, 2.5; 95% CI, 1.5–4.2; *P* = 0.0008), and DDFS (aHR, 2.9; 95% CI, 1.6–5.3; *P* = 0.0004) but was not associated with a poor prognosis in postmenopausal women (Figure 1). In contrast, haplotype *AASC* was significantly associated with a poor OS (aHR, 4.4; 95% CI, 1.4–14.1; *P* = 0.01) and a poor DFS (aHR, 3.1; 95% CI, 1.1–8.3; *P* = 0.03) in postmenopausal women and a poor OS in all women combined (aHR, 2.9%; 95% CI, 1.3–6.3; *P* = 0.008) but not in premenopausal women.

In premenopausal patients with adjuvant chemotherapy (high-risk group), haplotype *AASA* was significantly associated with a poor OS (aHR, 6.4; 95% CI, 2.1–19.0; *P* = 0.0009), DFS (aHR, 3.2; 95% CI, 1.5–6.8; *P* = 0.003), and DDFS (aHR, 4.5; 95% CI, 1.9–10.3; *P* = 0.0005) compared to those without haplotype *AASA* as the reference (Table 4).

## 4. Discussion

In the present study, we demonstrated that, in LN-negative, HR-positive breast cancer patients, premenopausal women carrying the *CYP19* genetic polymorphism haplotype *AASA* had a significantly poorer DDFS, DFS, and OS than those without haplotype *AASA*. Even in patients who received chemotherapy, haplotype *AASA* remained a negative prognostic factor for DDFS, DFS, and OS. However, in postmenopausal women, haplotype *AASA* was not associated with poor DDFS, DFS, or OS, whereas haplotype *AASC* was significantly associated with poor OS and DFS. 

SNP rs4646 of *CYP19*, located in the 3′ untranslated region, was recently found to be associated with circulating steroid hormone levels and with the objective response to the aromatase inhibitor letrozole in postmenopausal breast cancer [13, 14, 24]. In the present study, we observed that the combined high risk A/A + A/C alleles of *CYP19* polymorphism rs4646 were significantly associated with a poor DDFS (*P* < 0.05) and marginally associated with a poor OS (*P* = 0.06) and poor DFS (*P* = 0.07).

SNP rs1065779 of *CYP19*, which is located in intron 9, 53 base pairs upstream of exon 10, has been suggested to affect transcription or expression of aromatase [11] and to be associated with lower serum estrogen levels in nonsmall cell lung carcinoma patients [25]. In the present study, we observed an increased risk of the A allele in *CYP19* polymorphism rs1065779 in terms of a poor prognosis for DFS and DDFS in premenopausal patients and those receiving adjuvant chemotherapy.

The TTTA repeat *CYP19 *polymorphism has been reported to be associated with higher aromatase activity [26, 27], and increasing repeat length is associated with a good prognosis of breast cancer [7, 8]. These observations are consistent with our findings, which also showed that an increased poor prognosis risk of DDFS, DFS, and OS was associated with the short TTTA repeat polymorphism in LN-negative, HR-positive breast cancer patients, especially premenopausal patients.

SNP rs1870050 of *CYP19*, located in the first exon close to promoter I.1, is associated with risk of endometrial cancer [12], and the cancer risk association is modified by tea consumption [11]. In the present study, we found that the A/A genotype of the *CYP19* polymorphism rs1870050 was associated with a poor DDFS in premenopausal women, but no significant associations were observed with OS or DFS. Further studies with larger sample sizes are needed to understand the effect of *CYP19* polymorphism rs1870050 on OS and DFS of LN-negative, HR-positive premenopausal breast cancer patients. 

In the present study, we found that, in premenopausal women, *CYP19 *polymorphisms rs4646 (A/C), rs1065779 (A/C), rs1870050 (A/C), and *CYP19 *(TTTA)n (S/S + S/L versus L/L) were significantly associated with a poor DDFS. Haplotypes derived from these 4 genetic polymorphisms confirmed the above association. We also found that premenopausal women carrying haplotype AASA not only had a poor DDFS but also a poor DFS and OS than those without AASA.

The poor prognostic effect of the *CYP19* haplotype AASA for premenopausal women is hypothesized to be due to differences in the regulatory mechanisms of estrogen between premenopausal and postmenopausal women. In breast cancer samples, expression of aromatase is upregulated in breast tumor tissue compared to normal tissue [28], indicating a potential role of *CYP19* in the development and progression of breast cancer, and microarray expression profiling and clustering analysis have demonstrated a significant positive correlation between aromatase and estrogen-related receptor *α* mRNA expression in isolated carcinoma cells [29]. In premenopausal women, estrogen is mainly produced by the ovary, while in postmenopausal women, aromatization of androgen in extragonadal tissue, for example, adipose tissue, is the main source [5]. It is therefore possible that the proliferation of HR-positive breast cancer cells in premenopausal women is more estrogen-dependent than that in postmenopausal woman and that antihormone treatment, such as tamoxifen and ovarian ablation, and chemotherapy, which also result in a menopausal status, might cause a greater decrease in ovary-synthesized estrogen to support breast cancer growth in premenopausal women. 

Given that cancers in premenopausal women may have a greater need of estrogen for growth than those in postmenopausal women, the tumor cells may grow rapidly and thus develop recurrence and distant metastases when they are in a high-estrogen microenvironment. We hypothesize that premenopausal women with haplotype AASA may have higher aromatase activity and have higher estrogen levels after antihormone therapy, since estrogen is still synthesized in adipose tissues by aromatase, and that this may explain why this group of patients has a poor survival. Our findings are in line with those of Long et al. [30] showing that haplotypes *CCCTA* (minor alleles for hCV1664178, rs12900137, rs730154, rs936306, and rs1902586 in block 2) and *AAGC* (nonsynonymous SNP rs727479, rs700519, rs10046, and rs4646 in block 4) are associated with poor DFS and OS in premenopausal women. 

Although the survival benefit of the non-*AASA* haplotypes of *CYP19* may be a result of a greater estrogen-reducing effect of tamoxifen in premenopausal women, we did not measure estrogen levels and their association with the *AASA* haplotype, since estrogen levels in premenopausal women do not remain constant because of their physiology. In addition, whether the *AASA* haplotype of* CYP19* is associated with the survival of postmenopausal women receiving an aromatase inhibitor needs to be examined. Furthermore, the significant association between haplotype *AASC* and poor OS and DFS observed in our postmenopausal women requires further studies to clarify the possible mechanism. 

In addition to germline SNPs of tamoxifen-metabolizing genes, such as *CYP19*, *CPY2D6, *and* SULT1A1* [8, 31–33], SNPs identified in genomewide association studies (GWAS) have been found to be associated with breast cancer risk [34, 35]. A recent study investigating the association between 8 risk SNPs identified in GWAS and breast cancer outcome demonstrated that rs12443621 (16q12) and rs6504950 (17q23) may influence OS and that patients with three or four at-risk genotypes of the GWAS SNPs have a higher risk of death than those with two or fewer at-risk genotypes [36]. Thus, further studies exploring the influence of candidate genes and GWAS-identified genes involved in metabolism of tamoxifen, chemotherapeutic agents, and estrogen on the effect of adjuvant hormonal therapy and the survival of HR-positive breast cancers are warranted. 

In conclusion, our results show that, in LN-negative, HR-positive breast cancer patients,* CYP19* genetic polymorphism haplotype *AASA* is associated with poor survival in premenopausal women but not in postmenopausal women. In high-risk premenopausal patients with adjuvant chemotherapy, haplotype *AASA* remains a negative prognostic factor, and new adjuvant treatment strategies for this group of patients are warranted. Clarification of the molecular mechanisms of the effect of haplotype *CYP19 *genetic polymorphism, such as mRNA stabilization, transcription enhancement, or posttranslational upregulation of aromatase expression [37], in this group of breast cancer patients is needed.

## Supplementary Material

Supplemental Table 1 shows three CYP19 SNPs, rs4646 in the 3' untranslated region, rs1065779 in the intro-exon boundary, and rs1870050 in the promoter region/untranslated exon carry a higher allelic frequency of CYP19 in Han Chinese from the data of Hapmap or NCBI.

## Figures and Tables

**Table 1:** Pertinent clinicopathologic features of the LN-negative, HR-positive breast cancer patients.

Characteristic	Number of patients (*N* = 296)
Age (years)	
Median (range)	50 (24–81)
HER-2/neu status	
0	65 (22.0)
1	62 (20.9)
2	38 (12.8)
3	41 (13.9)
Missing	90 (30.4)
Menopausal status	
Premenopausal	178 (60.1)
Postmenopausal	118 (39.9)
Pathology	
Infiltrating ductal ca. + infiltrating lobular ca. + medullary ca.	253 (85.5)
Other	43 (14.5)
Grade	
I	127 (42.9)
II	131 (44.3)
III	38 (12.9)
Tumor size (cm)	
≤2	171 (57.8)
>2–5	125 (42.2)
Hormone receptor status	
ER (+) PR (+)	220 (74.3)
ER (+) PR (−)	45 (15.2)
ER (−) PR (+)	31 (10.5)
Adjuvant hormone therapy	
Tamoxifen	269 (90.9)
Others*	27 (9.1)
Adjuvant chemotherapy	
No CT	162 (54.7)
CT	134 (45.3)

LN: lymph node; HR: hormone receptor; ca: cancer; ER: estrogen receptor; PR: progesterone receptor; CT: chemotherapy.

*Ovarian ablation or luteinizing hormone-releasing hormone.

**Table 2:** Association between various *CYP19 *genotypes and OS, DFS, or DDFS.

Genotype	OS		DFS		DDFS	
	Hazard ratio (95% CI) aHR (95% CI)	*P*	Hazard ratio (95% CI) aHR (95% CI)	*P*	Hazard ratio (95% CI) aHR (95% CI)	*P*
CYP19_rs4646 A/A + A/C versus C/C						
Total (*N* = 296)	1.6 (0.8–3.1) 1.8 (0.9–3.6)	0.17 0.09	1.6 (1.0–2.5) 1.6 (1.0–2.6)	0.06 0.06	1.6 (0.9–2.7) 1.7 (0.9–2.9)	0.10 0.07
Premenopausal (*n* = 178)	2.1 (0.9–4.9) 2.4 (1.0–5.9)	0.10 0.06	1.8 (1.0–3.1) 1.8 (0.9–3.2)	0.05 0.07	2.2 (1.1–4.4) 2.3 (1.1–4.9)	0.03 0.02
Postmenopausal (*n* = 118)	1.0 (0.3–2.9) 1.2 (0.4–3.7)	0.97 0.81	1.3 (0.5–3.0) 1.4 (0.6–3.5)	0.59 0.42	0.9 (0.3–2.2) 1.0 (0.4–2.7)	0.79 0.99
Premenopausal patients receiving adjuvant chemotherapy (*n* = 86)	2.2 (0.6–7.4) 2.8 (0.7–10.7)	0.22 0.13	1.8 (0.8–4.1) 1.7 (0.7–4.1)	0.15 0.24	2.1 (0.8–5.4) 2.2 (0.8–6.3)	0.12 0.13

CYP19_rs1065779 A/A + A/C versus C/C						
Total (*N* = 296)	1.3 (0.6–2.8) 1.6 (0.7–3.3)	0.50 0.26	2.0 (1.1–3.6) 2.3(1.2–4.2)	0.02 0.009	1.7 (0.9–3.2) 1.9 (1.0–3.7)	0.87 0.06
Premenopausal (*n* = 178)	2.2 (0.8–6.6) 2.6 (0.8–7.8)	0.15 0.10	3.1 (1.4–6.8) 3.5 (1.5–7.9)	0.006 0.003	2.9 (1.1–7.5) 3.2 (1.2–8.6)	0.03 0.02
Postmenopausal (*n* = 118)	0.5 (0.2–1.7) 0.7 (0.2–2.2)	0.29 0.49	1.0 (0.4–2.5) 1.1 (0.4–3.0)	0.93 0.84	0.7 (0.3–2.0) 0.9 (0.3–2.6)	0.56 0.84
Premenopausal patients receiving adjuvant chemotherapy (*n* = 86)	4.1 (0.5–31.8) 7.0 (0.8–62.6)	0.18 0.08	4.9 (1.1–20.7) 6.6 (1.5–29.4)	0.03 0.01	7.0 (0.9–52.0) 13.0 (1.6–104.8)	0.06 0.02

CYP19_(TTTA)n S/S + S/L versus L/L						
Total (*N* = 296)	2.8 (0.9–9.1) 3.3 (1.0–11.0)	0.09 0.05	2.4 (1.1–5.3) 2.8 (1.3–6.2)	0.03 0.01	2.4 (1.0–6.0) 2.8 (1.1–7.1)	0.06 0.03
Premenopausal (*n* = 178)	6.6 (0.9–48.7) 7.8 (1.0–58.9)	0.07 0.05	3.6 (1.3–10.1) 4.0 (1.4–11.4)	0.01 0.009	5.0 (1.2–20.7) 5.5 (1.3–23.5)	0.03 0.02
Postmenopausal (*n* = 118)	1.0 (0.2–4.5) 1.1 (0.2–5.1)	0.98 0.93	1.2 (0.3–4.0) 1.3 (0.4–4.8)	0.81 0.64	0.9 (0.3–3.1) 1.1 (0.3–4.1)	0.87 0.86
Premenopausal patients receiving adjuvant chemotherapy (*n* = 86)	— —		3.5 (0.8–15.1) 4.0 (0.9–17.9)	0.09 0.07	5.3 (0.7–39.9) 6.3 (0.8–50.8)	0.10 0.09

CYP19_rs1870050 A/A versus C/C + A/C						
Total (*N* = 296)	1.2 (0.6–2.3) 1.2 (0.6–2.4)	0.62 0.53	1.3 (0.8–2.1) 1.4 (0.8–2.2)	0.31 0.19	1.7 (1.0–2.9) 1.8 (1.0–3.1)	0.06 0.05
Premenopausal (*n* = 178)	1.7 (0.7–4.1) 1.9 (0.8–4.7)	0.22 0.14	1.4 (0.8–2.4) 1.5 (0.8–2.8)	0.28 0.17	1.9 (0.9–3.8) 2.1 (1.0–4.3)	0.07 0.05
Postmenopausal (*n* = 118)	0.6 (0.2–1.9) 0.6 (0.2–2.0)	0.41 0.42	1.1 (0.5–2.5) 1.0 (0.4–2.4)	0.86 0.99	1.3 (0.5–3.4) 1.2 (0.5–3.2)	0.54 0.70
Premenopausal patients receiving adjuvant chemotherapy (*n* = 86)	1.8 (0.5–6.9) 2.1 (0.5–8.5)	0.37 0.28	1.1 (0.5–2.6) 1.5 (0.6–3.7)	0.79 0.40	2.7 (0.9–8.1) 3.3 (1.0–10.7)	0.08 0.05

**Table 3:** Overall survival, disease-free survival, and distant disease-free survival of LN-negative, HR-positive breast cancer patients with the *CYP19 *AASA haplotype.

Haplotype Subject number = 214; Allele number = 428 (allelic frequency)	OS			DFS			DDFS		
	Event number (frequency)	Hazard ratio (95% CI) aHR (95% CI)	*P*	Event number (frequency)	Hazard ratio (95% CI) aHR (95% CI)	*P*	Event number (frequency)	Hazard ratio (95% CI) aHR (95% CI)	*P*
AASA versus non-AASA (17.1% versus 82.9%)									
Total	27 (10.3 %)	1.9 (1.1–3.5) 2.1 (1.2–3.9)	0.03 0.01	51 (23.8%)	1.9 (1.2–3.0) 2.0 (1.3–3.2)	0.004 0.002	41 (19.2%)	1.8 (1.1–3.0) 2.0 (1.2–3.2)	0.02 0.008
Premenopausal		2.9 (1.5–5.7) 3.3 (1.6–6.8)	0.002 0.001		2.4 (1.4–3.9) 2.5 (1.5–4.2)	0.001 0.0008		2.6 (1.5–4.6) 2.9 (1.6–5.3)	0.0009 0.0004
Postmenopausal		0.6 (0.1–2.7) 0.7 (0.2–3.4)	0.53 0.71		1.2 (0.5–2.9) 1.4 (0.6–3.6)	0.72 0.44		0.8 (0.3–2.2) 0.9 (0.3–2.6)	0.60 0.81

AASC versus non-AASC (8.9% versus 91.1%)									
Total	27 (10.3 %)	2.0 (0.9–4.2) 2.9 (1.3–6.3)	0.08 0.008	51 (23.8%)	1.3 (0.7–2.5) 1.5 (0.8–2.9)	0.36 0.22	41 (19.2%)	1.3 (0.6–2.5) 1.6 (0.8–3.4)	0.51 0.18
Premenopausal		1.5 (0.5–4.1) 2.1 (0.7–6.4)	0.48 0.18		1.0 (0.4–2.4) 1.1 (0.4–2.7)	0.93 0.86		1.1 (0.4–2.7) 1.4 (0.5–3.6)	0.89 0.54
Postmenopausal		3.3 (1.1–10.0) 4.4 (1.4–14.1)	0.04 0.01		2.2 (0.8–5.6) 3.1 (1.1–8.3)	0.11 0.03		1.7 (0.6–5.0) 2.5 (0.8–7.6)	0.31 0.10

CASA versus non-CASA (11.9% versus 88.1%)									
Total		0.6 (0.2–1.6) 0.6 (0.2–1.6)	0.30 0.31		0.8 (0.4–1.5) 0.8 (0.4–1.6)	0.48 0.58		0.8 (0.4–1.7) 0.8 (0.4–1.7)	0.62 0.58
Premenopausal		0.6 (0.2–2.1) 0.6 (0.2–2.1)	0.45 0.45		0.8 (0.3–1.7) 0.8 (0.4–1.8)	0.49 0.60		0.7 (0.3–1.8) 0.7 (0.3–1.7)	0.70 0.40
Postmenopausal		0.5 (0.1–3.5) 0.5 (0.1–4.1)	0.45 0.53		0.8 (0.3–2.7) 0.8 (0.2–2.7)	0.75 0.69		1.1 (0.3–3.8) 1.1 (0.3–4.2)	0.83 0.85

CCLA versus non-CCLA (41.1% versus 58.9%)									
Total		0.6 (0.3–1.1) 0.6 (0.3–1.0)	0.07 0.06		0.8 (0.5–1.2) 0.8 (0.5–1.2)	0.28 0.22		0.8 (0.5–1.3) 0.8 (0.5–1.2)	0.33 0.27
Premenopausal		0.5 (0.3–1.1) 0.6 (0.3–1.2)	0.10 0.14		0.7 (0.4–1.2) 0.8 (0.5–1.3)	0.22 0.30		0.7 (0.4–1.2) 0.7 (0.4–1.3)	0.20 0.28
Postmenopausal		0.7 (0.3–1.9) 0.6 (0.2–1.6)	0.46 0.33		0.9 (0.5–1.9) 0.8 (0.4–1.6)	0.88 0.49		1.0 (0.5–2.2) 0.8 (0.4–1.8)	0.91 0.64

CCLC versus non-CCLC (8.9% versus 91.1%)									
Total		0.8 (0.3–2.1) 0.6 (0.2–1.6)	0.62 0.28		0.6 (0.2–1.3) 0.4 (0.2–1.0)	0.18 0.05		0.5 (0.2–1.3) 0.4 (0.1–1.0)	0.14 0.05
Premenopausal		0.6 (0.1–2.3) 0.3 (0.1–1.5)	0.42 0.16		0.5 (0.2–1.5) 0.4 (0.1–1.2)	0.22 0.10		0.3 (0.1–1.4) 0.3 (0.1–1.1)	0.14 0.07
Postmenopausal		1.2 (0.3–5.4) 1.0 (0.2–4.5)	0.78 0.99		0.7 (0.2–2.7) 0.5 (0.1–2.4)	0.56 0.42		0.7 (0.2–3.1) 0.7 (0.2–2.9)	0.67 0.60

CCSA versus non-CCSA (7.5% versus 92.5%)									
Total		1.9 (0.9–4.2) 1.8 (0.8–4.0)	0.11 0.15		1.1 (0.5–2.2) 1.1 (0.5–2.4)	0.85 0.72		1.4 (0.7–2.9) 1.4 (0.7–3.0)	0.35 0.35
Premenopausal		1.8 (0.6–5.1) 1.6 (0.6–4.8)	0.27 0.37		1.1 (0.4–2.8) 1.0 (0.4–2.5)	0.81 0.99		1.4 (0.6–3.6) 1.3 (0.5–3.3)	0.44 0.63
Postmenopausal		2.1 (0.6–7.3) 1.8 (0.5–6.3)	0.23 0.36		1.1 (0.3–3.5) 1.1 (0.3–3.8)	0.93 0.85		1.4 (0.4–4.6) 1.4 (0.4–4.7)	0.59 0.61

**Table 4:** Haplotype AASA remains a significant prognostic factor for poor survival in premenopausal patients receiving adjuvant chemotherapy.

CYP19 haplotype AASA versus non-AASA	Hazard ratio (95% CI) aHR (95% CI)	*P* value
OS	5.1 (1.9–13.7) 6.4 (2.1–19.0)	*P *= 0.001 *P *= 0.0009

DFS	2.7 (1.3–5.4) 3.2 (1.5–6.8)	*P *= 0.005 *P *= 0.003

DDFS	2.9 (1.4–6.2) 4.5 (1.9–10.3)	*P *= 0.005 *P *= 0.0005

## Abbreviations

LN: Lymph node
HR: Hormone receptor
DDFS: Distant disease-free survival
DFS: Disease-free survival
OS: Overall survival
aHR: Adjusted hazard ratio
ER: Estrogen receptor
PR: Progesterone receptor
SNP: Single nucleotide polymorphism.

## References

[1] EBCTCG (2005). Effects of chemotherapy and hormonal therapy for early breast cancer on recurrence and 15-year survival: an overview of the randomised trials. *The Lancet*.

[2] Burstein HJ, Prestrud AA, Seidenfeld J (2010). American Society of Clinical Oncology clinical practice guideline: Update on adjuvant endocrine therapy for women with hormone receptor-positive breast cancer. *Journal of Clinical Oncology*.

[3] Zhao Y, Mendelson CR, Simpson ER (1995). Characterization of the sequences of the human CYP19 (aromatase) gene that mediate regulation by glucocorticoids in adipose stromal cells and fetal hepatocytes. *Molecular Endocrinology*.

[4] Bulun SE, Sebastian S, Takayama K, Suzuki T, Sasano H, Shozu M (2003). The human CYP19 (aromatase P450) gene: update on physiologic roles and genomic organization of promoters. *Journal of Steroid Biochemistry and Molecular Biology*.

[5] Santen RJ, Brodie H, Simpson ER, Siiteri PK, Brodie A (2009). History of aromatase: saga of an important biological mediator and therapeutic target. *Endocrine Reviews*.

[6] Haiman CA, Hankinson SE, Spiegelman D (2000). A tetranucleotide repeat polymorphism in *CYP19* and breast cancer risk. *International Journal of Cancer*.

[7] Goode EL, Dunning AM, Kuschel B (2002). Effect of germ-line genetic variation on breast cancer survival in a population-based study. *Cancer Research*.

[8] Huang C-S, Kuo S-H, Lien H-C (2008). The *CYP19* TTTA repeat polymorphism is related to the prognosis of premenopausal stage I-II and operable stage III breast cancers. *Oncologist*.

[9] Ma CX, Adjei AA, Salavaggione OE (2005). Human aromatase: gene resequencing and functional genomics. *Cancer Research*.

[10] Paynter RA, Hankinson SE, Colditz GA, Kraft P, Hunter DJ, De Vivo I (2005). *CYP19* (Aromatase) haplotypes and endometrial cancer risk. *International Journal of Cancer*.

[11] Xu WH, Dai Q, Xiang YB (2007). Interaction of soy food and tea consumption with CYP19 A1 genetic polymorphisms in the development of endometrial cancer. *American Journal of Epidemiology*.

[12] Meng HT, Cai Q, Zhang Z-F (2007). Polymorphisms in the CYP19A1 (aromatase) gene and endometrial cancer risk in Chinese women. *Cancer Epidemiology Biomarkers and Prevention*.

[13] Colomer R, Monzo M, Tusquets I (2008). A single-nucleotide polymorphismin the aromatase gene is associated with the efficacy of the aromatase inhibitor letrozole in advanced breast carcinoma. *Clinical Cancer Research*.

[14] Garcia-Casado Z, Guerrero-Zotano A, Llombart-Cussac A (2010). A polymorphism at the 3’-UTR region of the aromatase gene defines a subgroup of postmenopausal breast cancer patients with poor response to neoadjuvant letrozole. *BMC Cancer*.

[15] Clark AG (2004). The role of haplotypes in candidate gene studies. *Genetic Epidemiology*.

[16] Schaid DJ (2004). Evaluating associations of haplotypes with traits. *Genetic Epidemiology*.

[17] Olson JE, Ingle JN, Ma CX (2007). A comprehensive examination of CYP19 variation and risk of breast cancer using two haplotype-tagging approaches. *Breast Cancer Research and Treatment*.

[18] Kuo S-H, Lien H-C, You S-L (2008). Dose variation and regimen modification of adjuvant chemotherapy in daily practice affect survival of stage I-II and operable stage III Taiwanese breast cancer patients. *Breast*.

[19] Goldhirsch A, Ingle JN, Gelber RD, Coates AS, Thürlimann B, Senn H-J (2009). Thresholds for therapies: highlights of the St Gallen international expert consensus on the primary therapy of early breast cancer 2009. *Annals of Oncology*.

[20] Lu Y-S, Kuo S-H, Huang C-S (2004). Recent advances in the management of primary breast cancers. *Journal of the Formosan Medical Association*.

[21] Kaufmann M, Morrow M, Von Minckwitz G, Harris JR (2010). Locoregional treatment of primary breast cancer: consensus recommendations from an international expert panel. *Cancer*.

[22] Tan S-H, Lee S-C, Goh B-C, Wong J (2008). Pharmacogenetics in breast cancer therapy. *Clinical Cancer Research*.

[23] Hudis CA, Barlow WE, Costantino JP (2007). Proposal for standardized definitions for efficacy end points in adjuvant breast cancer trials: the STEEP system. *Journal of Clinical Oncology*.

[24] Haiman CA, Dossus L, Setiawan VW (2007). Genetic variation at the CYP19A1 locus predicts circulating estrogen levels but not breast cancer risk in postmenopausal women. *Cancer Research*.

[25] Olivo-Marston SE, Mechanic LE, Mollerup S (2010). Serum estrogen and tumor-positive estrogen receptor-alpha are strong prognostic classifiers of non-small-cell lung cancer survival in both men and women. *Carcinogenesis*.

[26] Kristensen VN, Harada N, Yoshimura N (2000). Genetic variants of CYP19 (aromatase) and breast cancer risk. *Oncogene*.

[27] Gennari L, Masi L, Merlotti D (2004). A polymorphic CYP19 TTTA repeat influences aromatase activity and estrogen levels in elderly men: effects on bone metabolism. *Journal of Clinical Endocrinology and Metabolism*.

[28] de Jong PC, Blankenstein MA, van de Ven J, Nortier JWR, Blijham GH, Thijssen JHH (2001). Importance of local aromatase activity in hormone-dependent breast cancer: a review. *Breast*.

[29] Miki Y, Suzuki T, Tazawa C (2007). Aromatase localization in human breast cancer tissues: possible interactions between intratumoral stromal and parenchymal cells. *Cancer Research*.

[30] Long J-R, Kataoka N, Shu X-O (2006). Genetic polymorphisms of the CYP19A1 gene and breast cancer survival. *Cancer Epidemiology Biomarkers and Prevention*.

[31] Goetz MP, Rae JM, Suman VJ (2005). Pharmacogenetics of tamoxifen biotransformation is associated with clinical outcomes of efficacy and hot flashes. *Journal of Clinical Oncology*.

[32] Falany CN, Wheeler J, Oh TS, Falany JL (1994). Steroid sulfation by expressed human cytosolic sulfotransferases. *Journal of Steroid Biochemistry and Molecular Biology*.

[33] Huang C-S, Lin C-H, Lu Y-S, Shen C-Y (2010). Unique features of breast cancer in Asian women-Breast cancer in Taiwan as an example. *Journal of Steroid Biochemistry and Molecular Biology*.

[34] Easton DF, Pooley KA, Dunning AM (2007). Genome-wide association study identifies novel breast cancer susceptibility loci. *Nature*.

[35] Nordgard SH, Johansen FE, Alnæs GIG, Naume B, Børresen-Dale A-L, Kristensen VN (2007). Genes harbouring susceptibility SNPs are differentially expressed in the breast cancer subtypes. *Breast Cancer Research*.

[36] Bayraktar S, Thompson PA, Yoo SY (2013). The relationship between eight GWAS-identified single-nucleotide polymorphisms and primary breast cancer outcomes. *The Oncologist*.

[37] Chen S, Ye J, Kijima I, Kinoshita Y, Zhou D (2005). Positive and negative transcriptional regulation of aromatase expression in human breast cancer tissue. *Journal of Steroid Biochemistry and Molecular Biology*.

