# Supplementary material for: CYP19 Genetic Polymorphism Haplotype AASA Is Associated with a Poor Prognosis in Premenopausal Women with Lymph Node-Negative, Hormone Receptor-Positive Breast Cancer
Source: Biomed Res Int. 2013 Nov 14;2013:562197. doi: 10.1155/2013/562197 (PMC3845431; doi:10.1155/2013/562197)
Supplement: Supplementary file 1 — Supplemental Table 1 shows three CYP19 SNPs, rs4646 in the 3' untranslated region, rs1065779 in the intro-exon boundary, and rs1870050 in the promoter region/untranslated exon carry a higher allelic frequency of CYP19 in Han Chinese from the data of Hapmap or NCBI. [file 562197.f1.pdf]

Supplemental Table 1. Allelic frequency of CYP19 in Hapmap or NCBI

Marker<br>Allelic frequency	CEU[a]	CHB[b]
rs4646 C/A	0.77/0.23	0.70/0.30
rs1065779 C/A	0.58/0.42	0.53/ 0.47
rs1870050 A/C	0.91/0.09	0.77/0.23
(TTTA)n	0.63/0.37	0.75/0.25

NCBI: National Center for Biotechnology Information
[a]CEU: Utah residents with Northern and Western European ancestry from the CEPH collection
[b]CHB: Han Chinese in Beijing, China
